# Supplementary material for: Mapping the evolution of fertility support policies in China: A content and instrumental analysis
Source: PLoS One. 2025 Oct 9;20(10):e0332137. doi: 10.1371/journal.pone.0332137 (PMC12510515; doi:10.1371/journal.pone.0332137)
Supplement: S1 Appendix — (ZIP) [file pone.0332137.s001.zip › S1 Appendix. 226 original policy documents/61-国务院办公厅关于印发人口发展“十一五”和2020年规划的通知(FBM-CLI-2-88392).doc]

国务院办公厅关于印发人口发展“十一五”和2020年规划的通知

**发布部门：** [国务院办公厅](https://www.pkulaw.com/law/chl?Aggs.IssueDepartment=601) [机构沿革](javascript:void(0))

**发文字号：**国办发〔2006〕107号

**发布日期：**2006.12.29

**实施日期：**2006.12.29

**时效性：** [现行有效](https://www.pkulaw.com/law/chl?Aggs.TimelinessDic=01)

**效力级别：** [国务院规范性文件](https://www.pkulaw.com/law/chl?Aggs.EffectivenessDic=XC0203)

**法规类别：** [人口计划管理](https://www.pkulaw.com/law/chl?Aggs.Category=09701)

国务院办公厅关于印发人口发展“十一五”和2020年规划的通知
（国办发〔2006〕107号）

各省、自治区、直辖市人民政府，国务院各部委、各直属机构：
　　《人口发展“十一五”和2020年规划》已经国务院同意，现印发给你们，请认真贯彻执行。

国务院办公厅
二○○六年十二月二十九日

人口发展“十一五”和2020年规划

　　根据《[中华人民共和国国民经济和社会发展第十一个五年规划纲要](https://www.pkulaw.com/chl/62f50194a99f4ce8bdfb.html)》和《[中共中央国务院关于全面加强人口和计划生育工作统筹解决人口问题的决定](https://www.pkulaw.com/chl/5e7b01010a4102ffbdfb.html)》精神，制定本规划，旨在阐明国家人口发展战略思路与目标，明确“十一五”时期人口发展的主要任务，动员社会资源，促进人口资源的优化配置，为全面建设小康社会和构建社会主义和谐社会创造良好的人口环境，为经济社会全面协调可持续发展提供持久动力和增长源泉。
　　**一、**“十五”时期人口发展成就与经验
　　“十五”时期，在党中央、国务院的正确领导下，经过全国人民的共同努力，我国人口发展取得巨大成就。
　　--低生育水平继续保持稳定。坚持计划生育基本国策不动摇，总和生育率稳定在1.8左右，顺利实现了“十五”人口总量控制目标。实行计划生育以来，全国少生4亿多人，有效地缓解了人口对资源、环境的压力，有力地促进了经济发展和社会进步。
　　--人口素质不断改善。积极推进科教兴国战略和人才强国战略，科技、教育、文化、卫生等事业不断发展。国民平均受教育年限继续提高，15岁以上人口平均受教育水平提高到8.5年左右。婴儿死亡率、孕产妇死亡率有所下降，人口平均预期寿命2005年底达71.95岁。
　　--就业与社会保障水平不断提高。实施积极就业政策，就业总量稳步增长，就业结构进一步优化，就业局势保持基本稳定。城镇社会保障覆盖面继续扩大，农村社会保障制度建设取得积极进展。农村计划生育家庭奖励扶助制度和“少生快富”工程试点成效显著。
　　--城镇化水平进一步提高。积极引导农村富余劳动力转移就业和人口在城乡间、区域间的迁移，“十五”时期累计转移农业劳动力4000万人。2005年城镇化率达43%。
　　--人权事业取得新进展。“国家尊重和保障人权”载入[宪法](https://www.pkulaw.com/chl/b62e1b65233709b3bdfb.html)。努力缩小贫富差距，妇女、儿童、老年人和残疾人的权益保护进一步加强。提前完成了联合国千年发展目标中将贫困人口减半的目标。集中解决了“八七”扶贫攻坚计划中剩余的2423万农村人口饮水困难问题。
　　人口发展的基本经验是：坚持以邓小平理论和“三个代表”重要思想为指导，全面贯彻落实科学发展观，用发展和改革的办法研究解决人口发展中出现的新情况、新问题；坚持马克思主义人口理论与中国实际相结合，努力探索适应社会主义市场经济体制要求的具有中国特色的统筹解决人口问题的道路；坚持发展经济与控制人口数量、提高人口素质相结合，长期实行计划生育和男女平等基本国策，实施科教兴国战略、人才强国战略和可持续发展战略，促进人口与经济、社会、资源、环境协调和可持续发展；坚持党政第一把手亲自抓、负总责，充分发挥党和政府在人口发展领域的主导作用；坚持以人为本，以人的全面发展为中心，积极推进理论创新、体制创新、管理创新和科技创新，不断提高社会管理和公共服务水平；坚持人口发展领域的对外开放和国际合作，树立负责任人口大国的良好形象。

　　**二、**全面建设小康社会面临的人口形势
　　21世纪上半叶，在稳定低生育水平的前提下，我国将先后迎来劳动年龄人口、总人口和老年人口三大高峰。全面建设小康社会时期，我国人口形势依然严峻，人口发展面临着前所未有的复杂局面，人口与资源环境的矛盾日趋尖锐，人口安全问题不容忽视。人口数量、素质、结构和分布问题相互交织、相互影响，将对经济社会全面协调可持续发展产生重大影响，并直接关系到全面建设小康社会目标的实现。
　　（一）人口总量大且持续增长，低生育水平不稳定。
　　未来十几年我国总人口（不含香港、澳门特别行政区和台湾省，下同）每年仍将净增800万人-1000万人。全面建设小康社会，实现人均国内生产总值达到3000美元左右的目标，总和生育率必须稳定在1.8左右。按此预测，2010年和2020年的人口总量将分别达13.6亿人和14.5亿人；人口总量高峰将出现在本世纪30年代，达15亿人左右。
　　受20世纪80年代-90年代第三次出生人口高峰的影响，未来十几年，20岁-29岁生育旺盛期妇女数量将形成一个小高峰；9000多万独生子女陆续进入生育年龄，政策内生育水平将有所提高。上述两方面的共同作用，将使出生率有所回升，出生人口数有所增加。
　　部分群众生育意愿与现行生育政策要求还存在差距，中西部地区生育水平明显高于东部地区，部分农村地区和流动人口计划外生育问题仍比较严重，低生育水平面临反弹的现实风险。
　　（二）人口素质总体不高，制约社会主义和谐社会的构建和综合国力的提高。
　　健康素质较低。由生物、社会、行为与环境综合所致的出生缺陷发生率为4%-6%，个别地区达8%以上。据估计，全国每年约有20万-30万肉眼可见先天畸形儿出生，加上出生后逐渐显现出来的缺陷，先天残疾儿童总数达80万人-120万人，直接影响近百万个家庭、几百万人的生产和生活，并给全社会带来难以估量的损失和负担。各种地方病、传染病严重威胁人民群众身体健康。全国现有智力残疾人约544万人，精神障碍患者约1600万人。乙肝病毒携带者达1.2亿人，艾滋病开始从高危人群向一般人群扩散。
　　科学文化素质总体不高。我国人均受教育程度相对较低。2000年第五次人口普查结果显示，全国15岁以上文盲达8507万人，农村15岁-64岁劳动年龄人口小学以下文化程度的占47.6%，25岁-64岁人口中具有大专以上文化程度的仅为4.63%。目前，技师和高级技师仅占技能劳动者总量的4%，且一半以上技师和高级技师年龄在45岁以上。2005年我国每万人中科学家和工程师仅有17人。
　　道德素质建设亟待加强。部分人群心理失衡，道德失范，诚信缺失，社会责任感缺乏。
　　（三）人口结构性矛盾日益突出，对经济社会发展影响深远。
　　人口老龄化进程加快。2005年，我国65岁以上老年人口比重达7.7%以上，60岁以上老年人口比重达11.0%以上；到2020年，预计将分别达11.2%和16.1%，老年人口分别为1.64亿人和2.34亿人。到本世纪中叶，预计65岁以上老年人口将占总人口的1/4。预计80岁以上老年人口到2020年达2200万人，到2050年达8300万人。老年人口规模日益增大，对经济社会发展将产生重大影响。受人口流动迁移影响，农村老龄问题更为严重。
　　出生人口性别比居高不下。据统计，全国出生人口性别比2000年为116.86，2005年为118.58，严重偏离正常范围。到2020年，预计20岁-45岁男性将比女性多3000万人左右。出生人口性别比过高、持续时间过长，将影响社会的稳定与和谐。
　　劳动年龄人口数量庞大且保持增长态势。据预测，到2016年，15岁-64岁劳动年龄人口将达到峰值10.1亿人，到2020年达10亿人左右；15岁-59岁劳动年龄人口将于2013年达到峰值9.32亿人，到2020年达9.30亿人左右。庞大的劳动年龄人口，使扩大就业和减少失业成为当前和今后长时期重大而艰巨的任务。
　　区域间人口发展差异明显。农村、中西部和贫困地区面临生育水平较高、人口自然增长率较高、文盲率高、孕产妇死亡率较高、平均受教育年限较短等问题，留守老人、儿童的照料和教育问题突出；城镇与农村人口社会保障水平不平衡。城乡间、地区间收入差距拉大的趋势没有明显改善，由此引发的各种深层次社会矛盾严重影响社会主义和谐社会的构建。
　　（四）流动迁移人口规模庞大，对社会管理和公共服务提出严峻挑战。
　　目前，我国农村富余劳动力仍有1.5亿人-1.7亿人。随着工业化和城镇化进程的加快，将有大量农村人口转为城镇人口。庞大的流动迁移人口在积极促进经济社会发展的同时，也对基础设施、资源环境尤其是政府的社会管理和公共服务构成巨大压力。流动人口的技能培训、就业、子女受教育、医疗卫生、社会保障及人口和计划生育管理与服务等难度加大，流动人口基本权益保障亟待加强。
　　（五）人口与资源、环境的矛盾日趋尖锐，促进可持续发展的机制亟待建立健全。
　　资源瓶颈约束趋紧。未来十几年，通过采取有效措施，我国供水总量能够满足人口增长和城镇化发展的需求，但城市供水水源结构单一，供水保障率不高，干旱年份缺水严重，农村人口不能使用清洁饮用水问题还未解决，确保城乡供水安全的任务依然艰巨。人均耕地面积不断下降，粮食需求量持续增长，对农业的综合生产能力提出更高的要求。
　　生态环境形势严峻。生态环境恶化的趋势尚未得到有效控制，森林资源破坏严重、草原退化、土地沙化和石漠化、水土流失严重、自然湿地萎缩。今后一个时期，环境面临越来越大的压力，污染将向农村蔓延。生态脆弱地区难以承载人口的不断增长，人居环境质量改善的难度加大。
　　全面建设小康社会时期是经济社会发展的重要战略机遇期，也是社会抚养比相对较低、人力资源丰富、储蓄率较高的人口红利期。应对复杂的人口形势，必须以科学发展观统领新时期人口工作全局，明确国家人口发展战略，用改革的思路和发展的办法解决前进中遇到的困难和问题。

　　**三、**“十一五”时期和2020年人口发展的指导思想、原则和目标
　　（一）指导思想。
　　以邓小平理论和“三个代表”重要思想为指导，全面贯彻落实科学发展观，按照构建社会主义和谐社会的要求，坚持以人为本，推进制度创新，优先投资于人的全面发展。稳定低生育水平，提高人口素质，改善人口结构，引导人口合理分布，保障人口安全。促进人口大国向人力资本强国的转变，促进人口与经济、社会、资源、环境协调和可持续发展。
　　（二）基本原则。
　　坚持用人的全面发展统筹解决人口数量、素质、结构与分布问题。坚持以人为本，实现好、维护好、发展好广大人民群众的根本利益，在稳定低生育水平的前提下，把提高人口素质放在更加突出的位置。
　　坚持人口与发展综合决策。确立和发挥人口发展在经济社会发展中的基础地位和能动作用，协调人口与经济社会政策。把人口规划与就业规划、城市和村镇规划及土地利用总体规划、生态建设规划和资源发展利用规划等结合起来。
　　坚持人口问题的综合治理。综合运用法律、行政、教育、经济等手段，完善人口宏观调控体系和管理服务体系。加快政府职能转变，形成政府主导、部门协同、社会参与的人口工作新格局。
　　坚持分类指导。在不同的地区、不同的发展阶段，战略导向和政策重点应有所不同。
　　（三）人口发展目标。
　　“十一五”时期人口发展目标：人口总量控制在13.6亿人以内；人口素质明显提高；群众享有基本的医疗保健服务，出生缺陷发生率逐步降低，婴儿死亡率降到14.9‰，孕产妇死亡率降到40/10万；全面普及九年义务教育，国民平均受教育年限提高到9年左右；就业持续增长；社会保障体系比较完善；贫困发生率继续下降；出生人口性别比升高势头得到有效遏制；城镇化率达到47%左右；有效缓解城乡间、区域间差距扩大的势头；人居环境有所改善。
　　2020年人口发展目标：人口总量控制在14.5亿人左右；人口素质大幅度提高；群众普遍享有较好的医疗保健服务，出生缺陷发生率、婴儿死亡率、孕产妇死亡率持续下降；基本普及高中阶段教育，国民平均受教育年限达11年左右；就业比较充分；基本建立覆盖城乡居民的社会保障体系；贫困人口继续减少；出生人口性别比趋于正常；城乡间、区域间差距扩大的趋势得到扭转；人居环境质量明显提高。

　　**四、**“十一五”时期人口发展的主要任务
　　（一）综合运用经济社会发展政策，确保低生育水平稳定。
　　保持现行生育政策的稳定性、连续性。“十一五”时期，要坚持计划生育基本国策和稳定现行生育政策不动摇，党政第一把手亲自抓、负总责不动摇，稳定人口和计划生育工作机构、队伍不动摇，不断创新人口和计划生育工作体制、机制、手段和方法不动摇。进一步深化综合改革，坚持依法行政、思想政治教育与利益导向相结合，综合运用法律、行政、教育、经济等手段，建立健全依法管理、村（居）民自治、优质服务、政策推动、综合治理的长效工作机制。认真执行《[中华人民共和国人口与计划生育法](https://www.pkulaw.com/chl/252f7ce51132230bbdfb.html)》及相关法律法规。深入开展国策、国情、人口形势教育，加强人口和计划生育法制宣传，倡导科学、文明、进步的婚育观念，引导群众自觉实行计划生育。提高计划生育管理服务能力，改善计划生育家庭生产生活条件。高度重视并切实解决低生育水平面临的反弹风险，将总和生育率稳定在1.8左右，确保人口总量目标的实现。
　　建立和完善政府为主、社会补充的人口和计划生育利益导向政策体系。逐步形成以奖励扶助、困难救助、养老和医疗扶助等为主体的较为完整的人口和计划生育利益导向机制。全面推行农村计划生育家庭奖励扶助制度和“少生快富”工程，落实独生子女父母奖励、计划生育免费基本技术服务制度。积极探索建立独生子女伤残死亡家庭扶助、长效节育措施奖励、节育手术保险、城市计划生育夫妇年老一次性奖励等制度。对符合社会救助条件的计划生育家庭，通过城乡最低生活保障、医疗救助以及农村五保户供养、特困户生活救助等制度予以帮助。在就业培训、合作医疗、扶贫开发、宅基地划分、改水改厕、沼气应用、新技术推广等方面，制定和完善对计划生育家庭特别是农村独生子女和双女户家庭的优先优惠政策。按照资格确认、资金管理、资金发放、社会监督等职责分设原则，确保每一个符合条件的对象领到奖励扶助金和享受优惠政策。
　　建立健全稳定低生育水平的社会管理和公共服务体系。全面加强基层基础工作。形成特色鲜明的行政部门、服务机构、自治组织、群众团体目标一致、上下互动、信息共享、运转高效的科学管理格局。全面实施《“十一五”期间农村基层计划生育服务体系建设规划》，切实加强面向基层的服务体系建设。国家重点支持中西部县（市、区）、乡（镇）中心服务站和流动服务车建设。逐步形成以县级服务站为龙头、乡镇中心服务站为骨干、普通乡镇服务站为依托、村服务室为基础、流动服务车为纽带，人口计生和卫生部门密切合作、功能互补、资源共享的农村人口和计划生育技术服务体系。利用网络优势，面向健康、亚健康人群，履行宣传教育、技术服务、信息咨询、药具发放和人员培训等职能。实施“新农村新家庭计划”和“生育关怀行动”，开展计划生育、生殖健康及家庭保健等服务。建立人口和计划生育技术服务质量保证体系。确保法律法规规定的各项奖励优惠政策、县乡人口和计划生育技术服务机构基本建设和队伍建设、计划生育经常性工作、计划生育免费基本技术服务等经费的落实。
　　建立流动人口计划生育统一管理、优质服务新体制。将流动人口纳入流入地人口总数，实行以流入地为主的目标管理双向考核。完善流动人口计划生育管理机构和服务网络，配备必要的社区计划生育专（兼）职人员。在人口和计划生育工作领域，流入地要按照“属地化管理、市民化服务”的原则，将流动人口计划生育管理服务纳入经常性工作范围，提供与户籍人口同等的免费服务。把进城务工人员计划生育管理服务经费纳入各级财政预算。强化社区流动人口登记制度，实现流动人口信息适时变动、异地查询和跟踪管理。为流动人口办理经商、务工、购房、租房、社会保障等手续时，相关部门应与人口和计划生育部门密切配合，互通信息。在流动人口集中的社区、企业、集贸市场等成立计划生育协会。流出地要配合流入地，做好外出人员的宣传培训、免费办理婚育证明等相关工作。
　　（二）提高出生人口素质，着力解决人口结构性问题。
　　推行优生优育，防治出生缺陷。科学制定提高出生人口素质的规划和行动计划，加强出生缺陷干预能力建设，全面实施出生缺陷干预工程，实行定期评估、通报制度。大力宣传和普及预防出生缺陷科学知识，实施计划生育生殖健康促进计划，加强婚育咨询和指导，积极开展婚前和孕前保健、孕产期保健、产前筛查和诊断、产后访视、新生儿疾病筛查和康复等工作；促进住院分娩和母乳喂养，为贫困妇女提供必要的生育救助和安全接生。倡导科学婚检。对影响出生缺陷的生物遗传、社会环境、不良生活方式等重大危险因素进行研究、评估和干预。大力普及婴幼儿抚养和家庭教育的科学知识，开展婴幼儿早期教育。强化独生子女社会行为教育和培养。
　　加大综合治理力度，有效遏制出生人口性别比升高势头。建立党政负责、部门配合、群众参与的标本兼治工作机制。加强综合治理的过程评估和责任考核。深入开展“关爱女孩行动”、“婚育新风进万家”活动。以消除性别歧视为重点，广泛宣传男女平等、少生优生等文明婚育观念，普及保护妇女儿童权益的法律法规知识。制定有利于女孩健康成长和妇女发展的社会经济政策，促进男女平等就业和共同参与社会经济活动。对农村计划生育女儿户给予奖励，在扶贫济困、慈善救助、贴息贷款、就业安排、项目扶持中对计划生育女儿户予以倾斜，推动“幸福工程”、“春蕾计划”等社会公益活动。鼓励男到女家落户，依法保护妇女的宅基地、房屋等继承权和土地承包权等权益。严禁非医学需要的胎儿性别鉴定和选择性别的人工终止妊娠。建立B超检查和人工终止妊娠登记、孕情检测、孕产过程管理等制度。完善执业资格认证和B超使用准入制度。对终止妊娠药品和促排卵药品实行严格的处方管理。运用法律手段，严厉打击非法实施胎儿性别鉴定和选择性别人工终止妊娠的行为，依法严惩溺、弃、残害女婴和拐卖、绑架妇女儿童的犯罪活动和歧视、虐待生育女婴的妇女等违法行为，保护妇女儿童合法权益。探索建立跨地区、跨部门的出生性别监控系统和选择性别终止妊娠综合监管制度。实施举报制度，加强社会监督。
　　建立健全养老保障体系和老年社会服务体系，积极应对人口老龄化。制定和落实老龄事业发展战略规划和政策，把逐步建立覆盖城乡居民的养老保障制度作为社会保障体系建设的重点，加强社区老年服务机构和基础设施建设，到2010年，初步构建以居家养老为基础、社区服务为依托、机构照料为补充的养老服务体系。加强农村乡镇敬老院、老年活动中心和综合性老年福利服务中心建设，争取使其覆盖75%以上的乡镇。在农村探索建立多种形式的计划生育家庭养老保险制度。有条件的地方，可建立政府、集体和社会共同参与的养老服务机构。对生活不能自理的农村计划生育家庭老年父母，按规定提供适当补助。对军烈属、鳏寡及其他有特殊困难的老年人，按规定给予养老救助。在城市逐步完善社会统筹与个人账户相结合的基本养老保险制度，构建多层次的城镇养老保障体系。积极发展适合老年人特点的知识和经验密集型服务业，为老年人提供力所能及参与社会的机会。提高养老服务机构在城市规划中的比重，发展社区老年活动场所和服务设施，制定优惠政策，鼓励社会开办各种类型的养老服务机构。发扬敬老、养老、助老的良好社会风尚，积极探索和实施“爱心护理”等工程。从老年预防保健入手，倡导健康生活方式，营造出行安全和起居方便的环境。探索建立老年服务志愿者、照顾储蓄、长期护理保险等社会化服务制度。大力弘扬子女赡养、家庭养老和邻里互助的传统美德。加强舆论监督，对拒绝赡养或虐待父母的行为，追究法律责任。大力发展老龄产业，建立满足特殊需求的老年用品和服务市场。倡导终生学习、终生保健，促进老年人身心健康。
　　（三）坚持教育优先发展，充分开发人力资源。
　　全面实施素质教育。坚持德育为先，把社会主义核心价值体系纳入国民教育全过程，切实加强和改进中小学思想道德建设和大学生思想政治教育。进一步深化教育教学改革，倡导和组织学生参加生产劳动和社会实践活动，加强对学生创新精神和实践能力的培养。提高学生的健康素质和审美素养。改革和完善考试评价制度，探索建立教育质量监测和督导评估体系。加强学校教育、家庭教育和社会教育的融合，形成全社会推进素质教育的合力。
　　普及和巩固九年义务教育，大力发展职业教育，提高高等教育质量。2007年，完成西部地区“两基”攻坚任务；到2010年，全国初中三年保留率达到95%。强化政府对义务教育的保障责任，对农村地区学生免收学杂费，并对贫困家庭学生提供免费课本和寄宿生活补助。切实解决好适龄女童、残疾儿童、进城务工农民子女接受义务教育的问题。扩大中等职业教育招生规模。以就业为导向，深化职业教育教学改革，大力推行工学结合、校企合作和半工半读的职业培训和技能劳动者培养模式。加强职业教育基础能力建设，建立和完善家庭经济困难学生资助政策体系。推行职业资格证书制度，提高劳动者就业能力和工作能力。切实把高等教育发展的重点放到提高质量上来，注重增强学生的实践能力、创造能力和就业能力、创业能力。组织实施好高等教育“211工程”和“985工程”。探索建立和完善产学研相结合的良性机制。
　　提高农村劳动力转移就业能力，积极培育新型农民。组织实施好新型农民科技培训工程和农村劳动力转移培训阳光工程，充分利用和整合各种职业培训资源，加强农村劳动力转移培训、农民工技能提升培训和农业实用技术培训，大力培养技能型人才和农村实用人才。完善政府、企业和个人共同分担的培训经费投入机制，对参加职业培训且符合条件的农村劳动力按规定给予一定补贴。
　　构建中国特色社会主义现代化教育体系，推进学习型社会建设。依法加大政府对教育的投入力度，鼓励和引导社会、企业和公民出资办学，积极构建学习型社会的保障体系。深化教育管理体制改革，理顺政府、学校与社会之间的关系，激发教育发展的活力。促进和规范民办教育持续健康发展。推动中外合作办学。积极发展现代远程教育，以信息化带动教育现代化。努力提高学前教育和高中阶段教育的普及程度。发展成人教育和多样化的继续教育。
　　（四）统筹城乡、区域协调发展，引导人口有序流动和合理分布。
　　在主体功能区建设中引导人口自愿、有序、平稳流动。根据资源环境承载能力、现有开发密度和发展潜力，统筹考虑未来我国人口分布、经济布局、国土利用和城镇化格局，将国土空间划分为优化开发、重点开发、限制开发和禁止开发四类主体功能区。发挥市场机制在人力资源配置中的基础性作用，引导人口在四类功能区之间合理分布，逐步改变人口分布与经济分布和生态环境承载能力失衡的状况。鼓励在优化开发区有稳定就业和住所的外来人口定居落户，鼓励重点开发区在加快工业化、城镇化进程中积极承接人口流入，引导限制开发区、禁止开发区的人口向优化开发区、重点开发区逐步平稳有序转移。对生态脆弱地区、自然保护区和重要生态功能区，实施生态移民和异地扶贫政策。综合协调不同区域、不同部门有关人口流动和迁移的政策法规，促进统一执法和科学执法，为促进人口的合理迁移创造有利的制度环境。
　　在新型工业化、城镇化进程中引导人口合理集聚。坚持大中小城市和小城镇协调发展，在资源环境容量允许的范围内提高城镇综合承载能力，按照循序渐进、节约土地、集约发展、合理布局的原则，积极稳妥地推进城镇化，引导人口向城镇有序集聚。具备条件的区域，要统筹规划，以特大城市和大城市为龙头，发挥中心城市作用，培育若干新城市群，使之成为集聚产业、创造就业、吸纳人口的重要载体。鼓励农村人口进入中小城市和小城镇定居，合理控制特大城市人口规模的过快增长。
　　坚持“合理引导、公平对待、完善管理、搞好服务”的方针，为农村富余劳动力有序、合理流动提供保障。加快建立城乡劳动者平等的就业制度和城乡统一的劳动力市场。完善覆盖城乡的制度化、专业化和社会化就业管理服务组织体系。建立全国劳动力市场信息监测、分析与发布制度。统筹管理城乡劳动力资源及其职业培训和就业促进工作。深化流动人口管理服务体制改革。逐步建立城乡统一的人口登记制度，健全出生人口登记和生命统计制度，实行流动人口居住证制度。推进进城务工人员权益保护立法，进一步明确其应有的合法权益和法律保护措施，改善农民进城就业、定居的制度政策环境。认真解决流动人口在就业、就医、定居、子女入托入学等方面的实际困难，逐步将进城务工人员纳入社会保障体系。搞好农村土地流转规范管理与服务，免除转移就业农民的后顾之忧。探索建立以现居住地为主、现居住地和户籍地互相配合的管理和服务体系，促进流出地就业培训机构与流入地就业服务机构之间的对接，使流动人口有更多的机会参与社区建设，分享社区的公共资源。
　　研究解决资源型城市和资源枯竭型城市人口与发展问题。对资源型城市人口与发展问题，要加强规划和管理，实现稳步开发与有效保护相结合，研究产业转换新路子，实现可持续发展。积极推进资源枯竭型城市转型试点，培育壮大接续替代产业，加强环境治理、生态恢复、公共设施建设，实行以工代赈，安排就业困难人员再就业。
　　（五）发展公共卫生、妇女儿童和社会福利事业，促进社会和谐与公平。
　　构建以预防为主的公共卫生服务体系。到2008年，基本建立新型农村合作医疗制度，巩固和健全基层医疗卫生服务体系。深化城镇医疗卫生体制改革，坚持公立医院的公益性质，积极支持和引导社会力量对卫生领域的投资。大力发展社区卫生服务，构建以社区卫生服务为基础的新型城市卫生服务体系。坚持政事分开、管办分开、医药分开、营利性与非营利性分开的原则，加强医疗卫生服务监管，控制医药费用不合理增长。预防和控制重大传染性疾病、重大寄生虫病和地方病，到2010年，艾滋病病毒感染人数控制在150万人以内，所有血吸虫病流行县（市、区）达到疫情控制标准，95%以上的县（市、区）实现消除碘缺乏病目标。建立健全公共卫生应急处理体系。加强妇幼卫生工作，提供以服务对象为中心、全面、综合的妇幼保健服务，预防和减少出生缺陷和残疾。科学实施健康干预，加强健康教育，引导人们建立健康的生活和行为方式，减少危险因素。大力开展全民健身运动。
　　促进妇女儿童发展，保障妇女儿童合法权益。制定《[中华人民共和国妇女权益保障法](https://www.pkulaw.com/chl/5ea182c212029b5ebdfb.html)》配套规范性文件，认真贯彻《[中华人民共和国未成年人保护法](https://www.pkulaw.com/chl/703c15790dd704dfbdfb.html)》等法律法规。建立和完善多部门参与的社会化妇女维权机制，保障妇女平等受教育和平等就业等权利。实施妇女教育培训计划和适合女性的创业项目推介万里行活动。建设家长学校和家庭教育指导机构，帮助家长掌握科学的教子方法，提高家庭教育水平。坚持“预防为主、综合治理”的方针，积极动员全社会各方面力量，齐抓共管，从根本上预防和减少青少年违法犯罪；加大对弱势儿童群体的社会保障力度，使孤残儿童、流浪儿童、单亲家庭和特困家庭儿童、留守儿童得到更多的社会关怀和救助。全面实现《[中国妇女发展纲要（2001-2010年）](https://www.pkulaw.com/chl/37733dd7246afab4bdfb.html)》和《中国儿童发展纲要（2001-2010年）》的各项目标。
　　改善残疾人平等参与社会生活的物质条件和精神环境。完善残疾人法律法规体系，依法维护残疾人的合法权益。全面推进“人人享有康复服务”，使830万残疾人得到不同程度的康复，增强残疾人生活自理和社会适应能力。提高残疾人受教育水平，保障残疾人受教育的权利，为有就业需求的残疾人提供职业指导和职业培训。做好农村残疾人扶贫工作，将贫困残疾人纳入社会保障范围。活跃残疾人文化、体育生活。加强残疾人无障碍环境建设。倡导理解、尊重、关心、帮助残疾人的良好社会风尚。
　　建立健全与经济发展水平相适应的社会保障体系。实现资金来源多渠道、保障方式多层次、管理服务社会化。建立包括最低生活保障、农村五保供养、医疗救助、教育救助、住房救助、司法援助等制度在内的新型城乡社会救助体系。将常年生活困难且符合条件的人口全部纳入救助范围。切实解决被征地农民的就业与保障问题。努力减少农村贫困人口。以整村推进、劳动力转移培训和产业化扶贫为重点，加大农村地区扶贫开发力度。

　　**五、**保障措施
　　（一）进一步加强对人口工作的领导。
　　各级政府要从落实科学发展观和构建社会主义和谐社会重大战略思想的高度，把人口发展摆到重要位置。充分认识统筹解决人口问题的长期性、艰巨性、复杂性和紧迫性，把人口发展作为国民经济和社会发展的出发点、着力点和落脚点，切实做到认识、责任、措施、投入“四个到位”。统筹人口规划、人口政策及相关经济社会发展政策等的制定和实施，加强对出生、婚姻、死亡、流动、户籍、就业、教育、医疗、税收、社会保障等人口管理制度的统一协调，建立健全人口发展的宏观调控体系和人口工作的综合治理机制。把人口和计划生育工作作为社会主义新农村建设的重要组成部分。
　　进一步完善党政第一把手亲自抓、负总责的责任制度。把领导干部落实人口发展目标的情况作为衡量政绩和选拔奖惩的重要内容，对工作失职的要追究责任。按照客观公正、突出重点、注重实效、鼓励创新、简便易行的原则，不断完善考核评估方法。
　　（二）构建人口与发展综合决策支持系统。
　　深入开展人口发展战略研究。人口问题是对国民经济和社会发展具有根本性、长期性影响的重大问题。“十一五”时期，在国家人口发展战略研究取得重大成果的基础上，进一步开展对和谐社会、功能区划、老龄化、人口流动迁移等重大课题的研究，不断揭示人口数量、素质、结构、分布与经济、社会、资源、环境之间的变动规律，研究制定与落实科学发展观和构建社会主义和谐社会要求相适应的人口发展指标体系和宏观调控政策。创新人口理论与研究方法，建立健全人口发展战略研究的长效机制。
　　构建人口与发展综合决策支持系统和监测评估体系。创新人口信息采集的手段和方法，建立快速、科学的监测机制，提高信息的真实性和权威性。加强流动人口的信息化建设，实现流动人口信息适时变动、异地查询和跟踪管理。建立部门间人口信息共享机制，实现互联互通和动态更新。加强人口信息的有效整合和系统分析。加快人口宏观管理与决策信息系统建设。建立和完善面向公众的人口信息公共服务网。
　　（三）改革和加强人口发展的社会管理和公共服务体系。
　　完善人口发展的法律法规体系，全面推行依法行政。继续贯彻依法治国基本方略，加强教育、就业、社会保障、卫生、人口和计划生育、环境保护等领域的立法，为实现人口发展目标提供可靠的法律保障。加强法制宣传，加大人口发展领域的执法力度，有法必依、执法必严，维护人民群众的合法权益，尊重和保护人权，使各项工作步入法制化管理的轨道。实行政务公开，加强社会监督。
　　加快转变政府职能。加强政府在人口发展中的社会管理和公共服务职能。在发挥政府主导作用的同时，利用市场机制，打破行政性垄断，充分发挥社区、企业和非政府组织的作用，逐步建立政府监管、社会主办的管理体制。积极探索政府购买服务等方式，调动社会力量兴办社会事业。
　　（四）建立优先投资于人的全面发展的公共财政投入体制。
　　建立稳定的促进人的全面发展的财政投入保障机制。调整财政支出结构，进一步加大对基础教育、就业培训、社会保障、公共卫生、人口和计划生育的投入。人口和计划生育财政投入增长幅度要高于经常性财政收入增长幅度。各级财政要逐年增加投入，到2010年，全国人均人口和计划生育事业费在“十五”期末人均10元的基础上增加到22元，继续安排“十五”期间已将社会抚养费、乡（镇）统筹费纳入财政预算的人口和计划生育事业费支出人均8元，届时，各级财政投入人口和计划生育事业费达到人均30元。进一步加大对农村、中西部和贫困地区人口发展的政策支持和资金扶持力度。
　　利用经济手段，形成良性循环的社会投入机制。建立多渠道的筹资体制，鼓励民间捐资、社会募捐和国际捐赠，引导企业、家庭、个人等加大对人力资本的投入力度。
　　（五）建立人口发展规划监测与评估机制。
　　抓好规划的实施。地方各级人民政府和有关部门共同实施人口发展规划。各地区、各部门要面向社会、面向群众，广泛开展对人口战略思想、发展目标、主要任务、重大政策与重大工程的宣传，在全社会形成关心和参与规划实施的氛围。要以本规划为基本依据，制定和实施相关年度计划。要根据实际情况和职责范围，制定本地区、本部门的具体实施方案。要通过规划引领项目，依靠项目促进规划的实施。
　　加强规划监督评估。在国务院的统一领导下，人口计生委和有关部门共同对人口发展规划的实施进行监督和评估。加强规划实施的动态监测与跟踪分析，及时发现和解决规划执行过程中存在的问题。在“十一五”中期和期末对规划执行情况进行考核评估。地方各级人民政府要定期组织对规划实施情况的检查，促进规划的顺利实施。在规划监督评估中，要发挥群众和团体的积极作用。

© 北大法宝：（[www.pkulaw.com](https://www.pkulaw.com/)) 专业提供法律信息、法学知识和法律软件领域各类解决方案。北大法宝为您提供丰富的参考资料，正式引用法规条文时请与标准文本核对。
欢迎查看所有[产品和服务](https://www.pkulaw.com/helps/2.html)。法宝快讯：[如何快速找到您需要的检索结果？](https://www.pkulaw.com/helps/70.html)[法宝](https://www.pkulaw.com/helps/69.html)V6有何新特色？

扫描二维码阅读原文

**原文链接：**<https://www.pkulaw.com/chl/61ee7e0b7f430fdbbdfb.html>
